# Changes in the Liver Function and Hematological Parameters in Dengue Patients at a Tertiary Care Center: A Descriptive Cross-sectional Study

**DOI:** 10.31729/jnma.8616

**Published:** 2024-06-30

**Authors:** Asmita Pokhrel, Buddhi Pokhrel, Richa Bhattarai, Madhav Khanal, Laxman Pokhrel

**Affiliations:** 1 Department of Biochemistry, Nepal Medical College Teaching Hospital, Jorpati, Kathmandu, Nepal; 2 Department of Biochemistry, Universal College of Medical Sciences, Bhairahawa, Rupendehi, Nepal

**Keywords:** *complete blood count*, *dengue*, *Kathmandu*, *liver function test*

## Abstract

**Introduction:**

Liver is most commonly affected in dengue often resulting in changes in the liver function test parameters. Alterations in hematological parameters are also reported which could serve as early prognostic markers especially in resource limited settings where serological tests for the diagnosis of dengue is not available. This study aims to analyze liver function test and hematological parameter changes in dengue infected patients.

**Methods:**

A descriptive cross-sectional study was conducted from December 2022 to October 2023 in serologically dengue positive patients. Liver function parameters and blood parameters were analyzed from 220 patients. The purposive sampling technique was employed during the selection of participants.

**Results:**

Out of 220 study participants, 113 (51.36%) were males and 107 (49.64%) were females. The median age of the participants was 35 years (IQR: 26 - 48 years). Elevated serum AST and ALT levels were present in 121 (55%) and 80 (36.36%) of the participants respectively. Thrombocytopenia and leukopenia were observed in 92 (41.82%) and 88 (40%) of the study participants respectively. The median hemoglobin level was 14.4 (IQR: 13-15.47) g/dl. Low hemoglobin level was found in 31 (14.09%) participants. The median red blood cell count was 4.91 (IQR: 4.49 - 5.28) millions/mm3 with decreased red blood cell count noted in 27 (12.27%) participants.

**Conclusions:**

Increased serum transaminases levels, thrombocytopenia and leukopenia are common laboratory findings in dengue patients.

## INTRODUCTION

Dengue, transmitted by Aedes mosquitoes, is an escalating health problem. It is often self-limiting, asymptomatic however symptomatic individuals may experience flu-like symptoms, jaundice, and potentially life-threatening complications sometimes.^1^ Early diagnosis and appropriate management are pivotal, significantly reducing the fatality rates to below 1%.^2^

The virus affects multiple organ systems, mostly the liver often resulting in altered liver function test (LFT). Liver dysfunction usually manifests as increased serum transaminases and high bilirubin level depending on the severity of hepatocyte injury.^3-5^ Hematological parameters like complete blood count (CBC), white blood cells count (WBC), platelets count, etc. are also altered in dengue patients which could serve as early prognostic indicators, particularly in resource limited settings where serological tests for dengue are not available.^6,7^

The 2022 Dengue outbreak in Nepal, was the largest recorded.^8,9^ Dengue is now spreading to hilly regions of Nepal, with cases reported in all 77 districts.^10,11^ This study aims to analyze LFT and hematological parameter changes in dengue infected patients in a tertiary care center in Kathmandu.

## METHODS

Ethical approval (Ref. No.: 34-0791080) was obtained from the Nepal Medical College - Institutional Review Committee (NMC-IRC). A descriptive hospital based cross-sectional study was conducted in laboratory of NMC from December 2022 to October 2023. Patients aged 18 years and above with confirmed dengue serology NS1 positive, and/or IgM positive, and/or IgG positive were included and those with history of chronic liver disease, chronic kidney disease, blood disorders and pregnant women were excluded from the study.

A total of 1800 serological tests for dengue were performed during the study period and 322 cases were reported serologically positive. Among the 322 positive cases reported, 220 patients fulfilling the inclusion criteria were included in the study after obtaining their verbal consent. The purposive sampling technique was used for the selection of participants. Demographic and medical details were recorded at the time of blood sample collection. The LFT and CBC of the serologically positive patients were analyzed. Serum bilirubin, liver enzymes, total protein and albumin were estimated using the dry chemistry method in Johnsons and Johnsons Vitros 250 fully automated analyzer. Hematological parameters were analyzed by Sysmex XN-550 hematology analyzer. Serological tests for dengue were conducted using Bioline Diagnostics Dengue combo kit. The following reference ranges were used as per manufacturer's instructions: red blood cells (RBC) - 3.8-4.8 millions/mm3; WBC - 4,000-11,000 /mm3; differential leucocyte count (DLC): neutrophil 40-70%; lymphocyte 20-45%; monocyte 2-10%; eosinophil 1-6%; basophil 0-1%); platelets - 150,000- 450,000 /mm3; hemoglobin (Hb)- 13-18 gm/dl, packed cell volume (PCV) - 37-47%; total bilirubin - 0.2-1 mg/dl; direct bilirubin - <0.3 mg/dl; alanine transaminase (ALT) - 10-40 U/L; aspartate transaminase (AST) - <40 U/L for males & <35 U/L for females; alkaline phosphatase (ALP) 30-120 U/L; albumin - 3.5 -4.2 gm/dl; and total protein- 6.5-8.3 gm/dl. Hyper and hypo conditions were assigned for values higher and lower than the given reference ranges.

Data entry was done in Microsoft excel which were then analyzed using SPSS software. Numerical data were checked for normality using Shapiro-Wilk test. Since all the parameters deviated significantly from normal distribution, median and inter-quartile range were used to express the average values. Categorical data were expressed in both frequency and percentage.

## RESULTS

**Figure 1:**
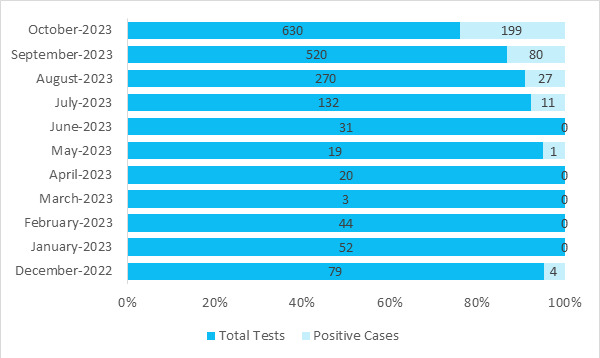
Month-wise prevalence of dengue positive cases.

Out of 1800 suspected cases, 322 (15.90%; 95% CI: 14.3%-17.5%) tested positive, 199 (61.80%) of positive cases were reported in month of October and 80 (26.66%) in September (Figure 1).

**Figure 2:**
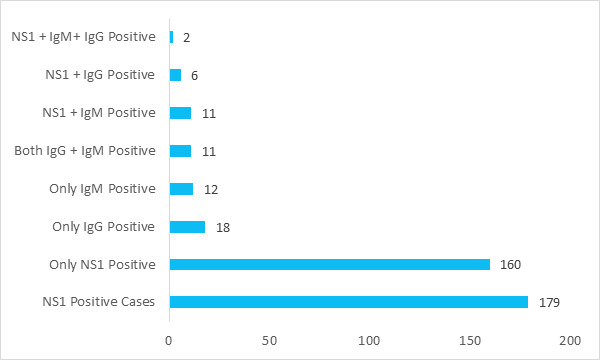
Serological details of the study participants.

Out of 220 study participants, 113 (51.36%) were males and 107 (48.64%) were females in our study. The median age of the participants was 35 years (IQR: 26 - 48 years). The majority of the participants ,179 (81.36%) tested positive for NS1. Among 220, patients, 40 (18.18%) and 37 (16.82%) patients were anti-dengue antibodies IgM and IgG positive respectively (Figure 2).

Elevated serum AST and ALT levels were present in 121 (55%) and 80 (36.36%) participants, respectively. Other LFT parameters were deranged in much lower percentage of the participants. Comprehensive details regarding LFT parameters (Table 1).

Among the CBC parameters, low platelets count and low WBC count were observed in 92 (41.81%) with median levels at 156,500 (IQR: 122,000 - 193,250) per mm^3^ and 88 (40%) with median levels at 4500 (IQR: 3300 - 6480) per mm^3^ of the study participants respectively. The median Hb level was 14.4 (IQR: 13-15.47) g/dl. Low Hb level was found in 31 (14.09%) participants. The median RBC count was 4.91 (IQR: 4.49 - 5.28) millions/mm^3^ with decreased RBC count noted in 27 (12.27%) participants (Table 2).

**Table 1:** Liver Function Test categories of the study participants (n= 220).

Parameters	Median	IQR	Low N (%)	Normal N (%)	High N (%)
Total Bilirubin (mg/dl)	0.60	0.53 - 0.78	-	198 (90)	22 (10)
Direct Bilirubin (mg/dl)	0.20	0.15 - 0.2	-	199 (90.45)	21 (9.54)
ALT (U/L)	33.3	22 - 63.05	-	140 (63.63)	80 (36.36)
AST (U/L)	40.2	29 - 72.32	-	99 (45)	121 (55)
ALP (U/L)	73.0	59.47 - 93.75	-	191 (86.81)	29 (13.18)
Albumin (gm/dl)	4.2	3.9 - 4.5	6 (2.72)	214 (97.27)	-
Total ProteOin (gm/dl)	6.8	6.4 - 7.2	20 (9.09)	200 (90.90)	-

Abbreviations: IQR: interquartile range, N: Number, %: percentages Hb: hemoglobin, RBC: red blood cells, TLC:total leucocytes count, PCV: packed cell volume

**Table 2:** Complete Blood Count (CBC) categories of the study participants (n= 220).

Parameters	Median	IQR	Low N (%)	Normal N (%)	High N (%)
Hb (gm/dl)	14.40	13-15.47	31 (14.09)	189 (85.90)	-
RBC (millions/mm3)	4.91	4.49 - 5.28	27 (12.27)	138 (62.72)	55 (25)
Platelets (cells/mm3)	156500	122000 - 193250	92 (41.81)	128 (58.18)	-
TLC (cells /mm3)	4500	3300 - 6480	88 (40)	124 (56)	8(4)
Neutrophils (%)	68	60 -75	-	123 (55.90)	97 (44.09)
Lymphocytes (%)	23	16 - 31	82 (37.27)	132 (60)	6 (2.72)
Eosinophils (%)	2	1 - 3	34 (15.45)	183 (83.18)	3 (1.36)
Monocytes (%)	6	4 - 9	-	192 (87.27)	28 (12.72)
Basophils (%)	0	0 - 0	-	218 (99.09)	2 (0.90)
PCV (%)	43.4	39.35 -46.65	22 (10)	156 (70.90)	42 (19.09)

Abbreviations: IQR: interquartile range, N: Number, %: percentages Hb: hemoglobin, RBC: red blood cells, TLC: total leucocytes count, PCV: packed cell volume

## DISCUSSION

Dengue now is endemic in Nepal with positive cases being reported throughout the year. Among 220 dengue positive patients, 113 (51.36%) were males and 107 (48.34%) were females which means males and females were affected almost equally. This is consistent with the findings of by Saud et al.^12^ However, Dhungana et al and Shrestha et al^14^ found males were affected slightly more than the females.^13^ Similar studies from other Asian countries also showed male preponderance.^15-17^

The median age of the participants in our study was 35 years. Similar mean age of the dengue patients were reported by Swamy et al (34.8 years)^5^, Shrestha et al (34.4 years)^14^ and Thapa et al (35.3 years)^18^, and while Dhungana et al,^13^ reported a lower median age of 29 years in his study. Dengue is common in younger adults, possibly due to their frequent travel and increased exposure to mosquito bites, given their active work lifestyles.^14^

Majority of the participants 179 (81%) in the present study were NS1 antigen positive (Figure 2). Similar results were shown by Saud et al^12^ (65%, 365), Shrestha et al^14^ (84%, 201), Thapa et al^18^ (90.5%, 76), who claimed NS1 antigen test as the most common serological positive tests among the dengue patients. This finding is in agreement with a study from Gujrat by Chhotala et al^19^ which reported 86% NS1 positive cases out of 100 infected patients. Higher number of NS1 antigen positive cases suggests that most of the patients presented during the active phase of the illness and active dengue infection circulating in the community.^18^ This alarms for the preparedness to prevent massive outbreaks in future and perform extensive epidemiological studies.

Regarding transaminases, our study found that more than a third 80 (36.36%) of participants exhibited raised ALT level, whereas AST levels were raised in more than half participants 121 (55%) (Table 1). Rise in serum transaminase levels were reported by Shrestha et al14 in more than 45% and by Thapa et al^18^ in more than 50% patients in their studies. Our findings closely align with the study conducted by Souza et al,^20^ which reported elevated ALT and AST levels in 45% and 63% of the infected patients, respectively. However, studies by Trung et al^21^ and Chhinaa et al^22^ reported much higher rates, with raised ALT and AST levels observed in over 90% of the infected patients. The mechanism for the rise in serum transaminases level in dengue patients may be attributed to reactive hepatitis or direct hepatocyte injury caused by the virus.^4^

In our study, 20 (9.09%) patients were detected with low serum total protein and 6 (2.72%) with low albumin (Table 1). Wong et al^17^ and Saha et al^23^ reported hypoalbuminemia among 16.5% and 12.9% dengue patients respectively which is slightly high number compared to our study. Whereas, Itha et al24 noted hypoalbuminemia in over two-third (76%) of the patients, a notable discrepancy from our study's observations. Hypoalbuminemia, which parallels disease severity, is primarily attributed to increased vascular permeability and plasma leakage.^3,4^

In our study, hyperbilirubinemia (high total bilirubin) was observed in 22 (10%) patients (Table 1). Wong et al17 reported 13.4% patients with elevated bilirubin, while Chhinaa et al^22^ and Saha et al^23^ documented rates of found 19.5 % and 16.9%, respectively. Furthermore, various studies mentioned above have depicted diverse prevalence rates of liver function abnormalities among dengue patients. They suggest that LFT levels fluctuate throughout the course of the illness. During the initial week, there is typically a greater elevation in AST compared to ALT, which then normalizes within three weeks of the infection.^3,4,5,22^ Additionally, AST levels tend to be higher across most studies, including ours, potentially attributed to the release of AST from extrahepatic sources such as the heart and striated muscles.^14^

While serological tests are crucial for definitive diagnosis of dengue, in regions where advanced biomedical infrastructures are lacking, CBC parameters emerge as pivotal early prognostic indicators for the disease.^6^ A prevalent trend entails progressive leukopenia, subsequent thrombocytopenia, and hemoconcentration, attributed to plasma leakage, particularly evident between days 3 to 8 of the illness. These hematological alterations in dengue patients may be ascribed to the direct suppression of bone marrow cells by the virus. Additionally, the virus intensifies peripheral destruction and immune-mediated clearance of platelets, exacerbating thrombocytopenia.^6,7^ In our study, thrombocytopenia 92 (41.81%) emerged as the most prevalent hematological abnormality, followed closely by leukopenia 88 (40%), (Table 2). These findings are consistent with studies conducted by Rao et al,^6^ and Ali et al.^25^ However, other studies from Nepal reported leukopenia as the commonest hematological finding followed by thrombocytopenia.^13,14,18^ Chaloemwong et al documented leukopenia and thrombocytopenia as the most common hematological abnormalities.^7^ These considerable discrepancies reported in percentages of leukopenia, thrombocytopenia, and DLC across the studies of the dengue patients are expected outcomes due to the ongoing dynamic changes in these parameters throughout the disease progression.^6,7,25^

The study was conducted at a single center, limiting the generalizability of our findings. Patients who were only IgG positive were also included in the study which could represent past infection. The day of fever onset or the timing of blood sampling were not recorded. The abscence of control group hinders our ability to make direct comparisons.

## CONCLUSIONS

Increased serum transaminases levels, thrombocytopenia and leukopenia are common laboratory findings in dengue patients.
